# Fatal meningitis in a previously healthy young adult caused by *Streptococcus pneumoniae *serotype 38: an emerging serotype?

**DOI:** 10.1186/1471-2334-5-38

**Published:** 2005-05-19

**Authors:** Carolyn I Baker, Christopher P Barrozo, Margaret AK Ryan, Lisa A Pearse, Kevin L Russell

**Affiliations:** 1 Department of Defense Center for Deployment Health Research, Naval Health Research Center, San Diego, California, USA; 2 Armed Forces Institute of Pathology, Rockville, Maryland, USA

## Abstract

**Background:**

In December 2001, a fatal case of pneumococcal meningitis in a Marine Corps recruit was identified. As pneumococcal vaccine usage in recruit populations is being considered, an investigation was initiated into the causative serotype.

**Case presentation:**

Traditional and molecular methods were utilized to determine the serotype of the infecting pneumococcus. The pneumococcal isolate was identified as serotype 38 (PS38), a serotype not covered by current vaccine formulations. The global significance of this serotype was explored in the medical literature, and found to be a rare but recognized cause of carriage and invasive disease.

**Conclusion:**

The potential of PS38 to cause severe disease is documented in this report. Current literature does not support the hypothesis that this serotype is increasing in incidence. However, as we monitor the changing epidemiology of pneumococcal illness in the US in this conjugate era, PS38 might find a more prominent and concerning niche as a replacement serotype.

## Background

In December 2001, the Department of Defense Center for Deployment Health Research at the Naval Health Research Center (NHRC) was consulted regarding a case of fatal meningitis caused by *Streptococcus pneumoniae *(pneumococcus) in a Marine Corps recruit.

The pneumococcus is a common cause of fatal bacterial meningitis in the US [1]. A 23-valent polysaccharide vaccine and a 7-valent conjugate pneumococcal vaccine are available and potentially effective in protecting against serotype-specific invasive infections. For this reason, it is important to determine whether clinically significant pneumococcal infections are vaccine-covered serotypes, particularly in settings like military training camps where epidemic spread can occur.

## Case presentation

On December 22, 2001, an 18-year-old male in his eighth week of Marine Corps basic training presented to the field medical station with headache and an episode of vomiting after physical training.

His neurological symptoms progressed over the next several hours until he became disoriented and unresponsive. He was evaluated in the local hospital emergency department where a fever of 39.1C (102.5F) and disorientation to person, place, and time were observed. Lumbar puncture performed during medical evaluation revealed an opening pressure greater than 500 mm; cerebrospinal fluid (CSF) was milky white, with a glucose concentration of 3 mg/dl and total protein of 269 mg/dl. White count in the CSF was 4444 with 91% segmented neutrophils, and microscopic evaluation of the CSF revealed Gram-positive diplococci. A presumptive diagnosis of pneumococcal meningitis was made; intravenous ceftriaxone, ampicillin, and vancomycin were initiated. Despite these measures, the patient's condition remained unimproved. He was transferred to the intensive care unit of a tertiary care hospital on ventilator support. Intravenous dexamethasone was initiated; antibiotics and supportive care were continued. He died approximately 30 hours after his initial presentation to the field medical station.

The patient had no previous history of meningitis, neurologic abnormalities or other medical problems. Prior to onset of his illness, there was no history of head trauma, and he was taking no medications. He received a meningococcal vaccination (Menomune A/C/Y/W135, Aventis Pasteur) on October 31, 2001. He had no history of receiving pneumococcal vaccination.

### Laboratory evaluation

Blood and CSF cultures were positive for *Streptococcus pneumoniae*, sensitive to all antibiotics tested. Streptococcal culture of the throat was negative.

A pneumococcal isolate from the blood was sent to the Respiratory Disease Laboratory at NHRC for additional evaluation. Serotyping was performed using a modified version of the latex agglutination typing method [2]. Samples were tested against antisera for vaccine-covered serotypes (Statens Serum Institut, Denmark) and results confirmed using the classic Quellung reaction and multi-locus sequence typing (MLST) technique.

For MLST, chromosomal DNA was extracted using spin DNA extraction columns (Qiagen, Valencia, CA). A subsequent PCR reaction was carried out in 100-μl volumes, using primer pairs developed by Enright and Spratt [3]. Each of the primer pairs amplify an internal fragment of 7 housekeeping genes: *aroE*, *gdh*, *gki*, *recP*, *spi*, *xpt*, and *ddl*. Amplified DNA fragments were purified using QIAquick purification columns (Qiagen, Valencia, CA) and directly sequenced in each direction on an automated sequencer with Big Dye terminator chemistry (Applied Biosystems, Foster City, CA). Allelic matches at each loci were determined through a search engine at the pneumococcal MLST Web site .

Using the classic serotyping methods outlined, the isolate did not react with any of the 23-valent vaccine-specific typing antisera. Molecular investigation utilizing MLST demonstrated an allelic profile that matched sequence type 393. Using the MLST database, this sequence type matched a likely pneumococcal serotype 38 (PS38). The latex agglutination was repeated using antisera against this serotype, and results were confirmatory.

PS38 is not covered by the current 23-valent polysaccharide or the 7-valent conjugate pneumococcal vaccine formulations, which include pneumococcal serotypes that are considered the most common causes of invasive and antibiotic-resistant infections in the US. Given the fatal outcome in this case and the importance of vaccine formulations being appropriately targeted, the medical literature was explored to evaluate pneumococcal disease caused by PS38 in the US and outside the US. Particular emphasis was placed on temporal trends and documented cases of meningitis caused by this serotype.

### PS38 in the US

Within the US, relatively few cases of PS38 disease have been reported. A few reported PS38 carriage isolates were found (3/235, 1.3%) from Alabama 1975 to 1978, but no cases of PS38 were among the 105 invasive isolates tested [4]. From 1985 to 1989 in Alabama, no PS38 was noted among 303 cases of meningitis or bacteremia, and only 2 cases were found among 228 cases of otitis media [5]. Black and colleagues reported two cases of invasive PS38 disease in children attending clinics in northern California from October 1995 to August 1998 [6]. A comprehensive analysis of pneumococcal isolates recovered from patients with invasive disease in the US in 1999 was performed by Gertz and colleagues. Among 1,168 isolates examined, only 5 were identified as PS38 (0.4%) [7]. More recently, three of 185 (1.6%) invasive isolates, and only 8/1379 (0.6%) carriage isolates were found to be PS38 among isolates from Alaska, 1998 to 2002 [8]. Given the invasive:carriage ratio of this and other analyzed studies, this report concluded that although the invasive *potential *of PS38 was similar to other vaccine serotypes, the carriage was so low that the significance of serious infections caused by PS38 was minimal at that time. Although not specified, meningitis was likely among these few documented invasive illnesses caused by PS38 within the US.

In a large review of the Active Bacterial Core Surveillance (ABCs) performed by Robinson and colleagues from 1995 to 1998, serotype 38 was not mentioned among the top 14 serotypes [9]. Likewise, numerous other studies failed to reveal PS38 among the reported contributing serotypes [1,10].

### PS38 outside the US

Cases of invasive PS38 disease have been reported in Australia, Spain, England, and Canada in recent years [11-16]. One Australian study identified 7 (12.4%) cases of invasive PS38 disease in non-indigenous adolescents, making PS38 the second most common non-vaccine covered serotype identified in this population [11]. Of particular interest is the study of pneumococcal isolates causing local and invasive disease in Spain from January 1997 to June 2001. Unlike the previous studies, a strain of PS38 was identified that was resistant to penicillin (MIC > 0.06 mg/L) [14].

In addition to causing invasive pneumococcal infections, PS38 has been found in isolates from children with acute otitis media in the Czech Republic [17] and from nasopharyngeal swabs in a study of pneumococcal carriage in Canadian children attending daycare from 1995 to 1996 [18]. In the study of pneumococcal carriage, PS38 ranked the 14th most common serotype, with 8 out of 589 (1.36%) isolates positive for PS38.

In contrast to these small studies with recognized PS38 disease or carriage, numerous other comprehensive reports from outside the US failed to demonstrate a burden. A review of over 70 global data sets performed by Hausdorff and colleagues failed to reveal PS38 among the top 25 serotypes identified among children; however, PS38 did rank #23 out of the top 24 isolates identified from adults [19]. In a paired report, Hausdorff did not assign significance to PS38 as he evaluated the disease manifestations of the top 16 global pneumococcal serotypes [20]. Likewise, in subsequent studies, PS38 was not mentioned among isolates of importance for otitis media or for invasive disease in the literature outside the US [8,21,22]. More recently, among invasive isolates considered: 150 from studies in Oxford, England, 1995–2001 [23]; 92 from Iceland, 1992–2001 [8]; 69 from Toronto, 1995 [24]; 112 from Kenya, 1992–1996 [25], and 56 from Papau New Guinea, 1981–1987 [26], PS38 was again not among the serotypes identified.

Among reports of studies outside the US that specifically address meningitis cases caused by PS38, Table 1 summarizes reports from Africa, Asia, Europe, and South America from the late 1970s to the mid-1990s. The prevalence of PS38-associated meningitis ranged from 0.2% in South Africa to 5.6% in Southern India. Of the cases with reported antibiotic sensitivities, all were penicillin-susceptible [27,28]. In four of the five reports, PS38 meningitis was among the 23 most common serotypes reported [28-31].

**Table 1:** Epidemiology of PS38 Meningitis Cases Outside the US. Studies outside the US of *Streptococcus pneumoniae *serotype 38 (PS38) isolated from the cerebrospinal fluid (CSF) of individuals being considered for the clinical diagnosis of meningitis.

**Location**	**Time Period**	**No. of Cases**	**Prevalence of PS38**	**Antibiotic Susceptibility**	**Patient Age Group**
**Cairo, Egypt [29]**	Late 1970s	3	2.3%	NP	Infants, Children, adults
**South Africa [27]**	1979–1986	2	0.2%	Pan-sensitive	NP
**São Paulo, Brazil [30]**	1977–1988	13	1.3%	NP	Children, adults, elderly
**Southern India [31]**	1992–1993	1	5.6%	NP	Children
**Netherlands [28]**	1994	3	2.0%	Penicillin-susceptible	NP

NP = Not provided

Of the PS38 disease reported outside the US, invasive pneumococcal disease was not uncommon. Additionally, even though most PS38 isolates tested were pan-sensitive to broad-spectrum antibiotics, antibiotics can be ineffective in a rapidly fulminating infection. This suggests considering primary measures such as vaccination for PS38 disease prevention. In some countries, PS38 was implicated in enough cases of invasive disease to be considered for inclusion into a 14- or 23-valent vaccine formulated for that region [28-31]. Although PS38 seems to be a sporadic cause of pneumococcal disease worldwide, temporal trends of increasing incidence were not appreciated.

## Conclusion

*Streptococcus pneumoniae *serotype 38 has been an uncommon cause of pneumococcal disease in the US and thus, is not included in commercial vaccine formulations. The significance of this serotype might be more important outside the US. Although the invasive potential of PS38 is documented, cases are rare. Prior to this report, a definitive meningitis death in the US caused by PS38 was not found in our review of the literature; nevertheless, there is little current evidence that PS38 is gaining in incidence or importance.

As we monitor the changing epidemiology of pneumococcal illness in the US in this conjugate era, PS38, with its documented invasive potential, might find a more prominent and concerning niche as a replacement serotype. Continued laboratory-based surveillance with a focus on emerging strains and the changing epidemiology is critical for optimal development of future prevention efforts in these dynamic times.

## List of abbreviations

NHRC – Naval Health Research Center

CSF – cerebrospinal fluid

MLST – multi-locus sequence typing

PS38 – pneumococcus serotype 38

ABC – Active Bacterial Core Surveillance

## Competing interests

The author(s) declare that they have no competing interests.

## Authors' contributions

CIB performed all coordination of sample processing, researched for the case report, and wrote the initial draft. CPB was responsible for all laboratory processing. MAKR provided oversight and expert consultation. LAP provided information on the clinical course of the patient during the hospital stay. KLR conceived of the case report, guided the laboratory processing and writing of the manuscript, and coordinated all aspects.

## Pre-publication history

The pre-publication history for this paper can be accessed here:


